# Recombinant Expression and Bioactivity Comparison of Four Typical Fungal Immunomodulatory Proteins from Three Main *Ganoderma* Species

**DOI:** 10.1186/s12896-018-0488-0

**Published:** 2018-12-14

**Authors:** Zheng-Wei Qu, Si-Ya Zhou, Shi-Xin Guan, Rui Gao, Zuo-Wen Duan, Xin Zhang, Wei-Yan Sun, Wen-Li Fan, Shui-Sen Chen, Li-Jing Chen, Jing-Wei Lin, Yan-Ye Ruan

**Affiliations:** 1 Liaoning Province Key Laboratory of Agricultural Technology, College of Bioscience and Biotechnology, Shenyang Agricultural University, Shenyang, 110866 China; 2 College of Horticulture, Shenyang Agricultural University, Shenyang, 110866 China

**Keywords:** *Ganoderma*, Fungal immunomodulatory protein, Recombinant expression, Cytotoxicity, Yeast

## Abstract

**Background:**

More than a dozen of fungal immunomodulatory proteins (FIPs) have been identified to date, most of which are from *Ganoderma* species. However, little is known about the similarities and differences between different *Ganoderma* FIPs’ bioactivities. In the current study, two FIP genes termed *FIP-gap1* and *FIP-gap2* from *G. applanatum*, along with *LZ-8* and *FIP-gsi*, another two representative *Ganoderma* FIP genes from *G. lucidum* and *G. sinense* were functionally expressed in *Pichia*. Subsequently, bioactivities of four recombinant *Ganoderma* FIPs were demonstrated and compared.

**Results:**

All the four *Ganoderma* FIP genes could be effectively expressed in *P. pastoris* GS115 at expression levels ranging from 197.5 to 264.3 mg L^− 1^ and simply purified by one step chromatography using HisTrap™ FF prepack columns. Amino acid sequence analysis showed that they all possessed the FIP conserved fragments. The homologies of different *Ganoderma* FIPs were from 72.6 to 86.4%. In vitro haemagglutination exhibited that FIP-gap1, FIP-gsi and LZ-8 could agglutinate human, sheep and mouse red blood cells but FIP-gap2 agglutinated none. Besides, the immunomodulation activities of these *Ganoderma* FIPs were as: rFIP-gap2 > rFIP-gap1 > rLZ-8 and rFIP-gsi in terms of proliferation stimulation and cytokine induction on murine splenocytes. Additionally, the cytotoxic activity of different FIPs was: rFIP-gap1 > rLZ-8 > rFIP-gsi > rFIP-gap2, examined by their inhibition of three human carcinomas A549, Hela and MCF-7.

**Conclusions:**

Taken together, four typical *Ganoderma* FIP genes could be functionally expressed in *P. pastoris*, which might supply as feasible efficient resources for further study and application. Both similarities and differences were indeed observed between *Ganoderma* FIPs in their amino acid sequences and bioactivities. Comprehensively, rFIP-gaps from *G. applanatum* proved to be more effective in immunomodulation and cytotoxic assays in vitro than rLZ-8 (*G. lucidum*) and rFIP-gsi (*G. sinense*).

**Electronic supplementary material:**

The online version of this article (10.1186/s12896-018-0488-0) contains supplementary material, which is available to authorized users.

## Background

*Ganoderma*s belong to *Ganodermataceae*, *Ganodermatales*, *Basidiomycetes* and Kingdom Fungi, which have been recorded for use in Orient over two millennia [1]. *Ganoderma*s have been believed to promote human’s health and longevity by treating very heavy diseases, and regarded as mysterious and auspicious herbs [2, 3]. Modern scientific studies have proven that *Ganoderma*s possess immunity-enhancing, anti-tumor, anti-allergy and anti-virus properties etc. [4–7] The bioactive components responsible for those activities include polysaccharides, glycopeptides, triterpenes, proteins, lectins, nucleotides, amino acids, sterols and alkaloids [8–12]. For example, three sterols and five triterpenes isolated from *G. annulare* were found to inhibit the growth of the fungi *Microsporum cannis* and *Trichophyton mentagrophytes* [13]. And polysaccharides from *G. lucidum* are extensively well-known for their anti-tumor and immunomodulation activities [14–16]. Recent studies exhibited that *G. lucidum* polysaccharides could serve as regenerative therapeutic agents to treat cognitive decline associated with neurodegenerative diseases by promoting cognitive function and neural progenitor proliferation [17] whereas four spiro-lactone lanostane triterpenoids isolated from *G. calidophilum* showed moderate cytotoxic activity against K562, BEL7402, and SGC790 cell lines [18]. Generally, *Ganoderma* polysaccharides and triterpenes are considered as the main effective gradients. Nevertheless, more and more attention has been paid to bioactive proteins from *Ganoderma*s in recent years [19–22]. Of all those bioactive proteins, fungal immunomodulatory proteins (FIPs) are a novelly-identified protein family, which share some amino acid sequence similarity and immunological response action to immunoglobulins [23]. Ever since the first FIP, known as LZ-8 or FIP-glu, was isolated from the mycelia of *G. lucidum* by Kino et al. in last century [24], more than fifteen FIPs have been identified from different fungi to date [25–31]. Nine of those FIPs are from *Ganoderma* species including LZ-8 (FIP-glu, *G. lucidum*), LZ-9 (*G. lucidum*), FIP-gts (*G. tsugae*), FIP-gja (*G. japonicum*), FIP-gsi (*G. sinense*), FIP-gmi (*G. microsporum*), FIP-gas (*G. astum*), FIP-gbo (*G. boninense*), FIP-gat (*G. atrum*), FIP-gap1 and FIP-gap2 (both from *G. applanatum*) [32–35]. The FIPs consist of 110–114 amino acids with molecular weights of 12–13 kDa, which are rich in Asp and Val but poor in His, Cys and Met. Different FIPs share some similarities in their amino acid sequences, for instance, LZ-8 and FIP-gts are 100% identical, whereas FIP-gmi, FIP-gsi, FIP-gat and FIP-gja share 99% similarities [28, 32]. Biological activities of FIPs have been extensively investigated since their discovery, mainly including hemagglutination, anti-anaphylaxis and anti-tumor [24, 25, 28, 30]. Recent studies revealed that FIP-fve exerted anti-inflammatory effects on OVA-induced airway inflammation by reducing airway remodeling and collagen expression [36]. And FIP-gmi could ablate cancer stemness and cisplatin resistance in oral carcinomas stem cells through IL-6/Stat3 signaling inhibition [37].

It is estimated that there are approximately 200 *Ganoderma* species around the world, among which more than 80 are identified in China [1–3]. Traditional classification of *Ganoderma*s in China was mainly made according to their morphological characteristics [38]. Zhao et al. divided *Ganoderma* into two main subgenera, *Ganoderma* and *Elfringia*. Subsequently, they found that subgenus *Ganoderma* contained two subsections, subsect. *Phaeonema* (Zizhi) and subsect. *Ganoderma* (Lingzhi) [39, 40]. Interestingly and coincidentally, FIPs have been identified from *G. lucidum* (*LZ-8* or *FIP-glu*, **Genbank No.** M58032.1) and *G. sinense* (*FIP-gsi*, **Genbank No.** AY449805.1), which are two most famous representative species from subsect. *Phaeonema* and subsect. *Ganoderma*. Similarly, a pair of novel FIP genes have also cloned from *G. applanatum*, the typical species of subgenus *Elfringia*, in our previous study [34, 35], which were termed as *FIP-gap1* (or known as *FIP-gap*, **Genbank No.** JN167598.1) and *FIP-gap2* (**Genbank No.** KX591653). However, very little is known about the exact differences and similarities between different *Ganoderma* FIPs until now. In the current study, those typical *Ganoderma* FIP genes from the three representative species were functionally expressed in *Pichia pastoris* GS115 and purified. Subsequently, a series of functional assays were performed using different recombinant *Ganoderma* FIPs in order to analyze and to compare their bioactivities comprehensively. Our results indicated that differences indeed existed between different *Ganoderma* FIPs despite of some similarities in their protein sequences and amino acid compositions. These findings will provide data on the biological functions of different *Ganoderma* FIPs which will help to elucidate the potential application and development of FIPs in biomedical or therapeutic studies.

## Results

### Sequence alignment and phylogenetic tree analysis

**Fig. 1:**
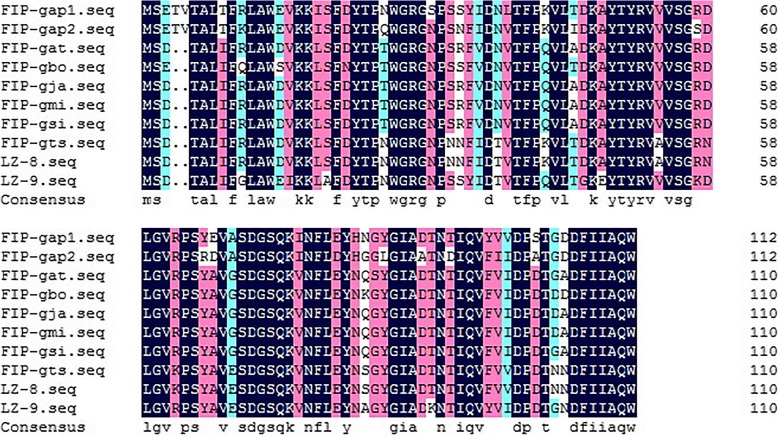
Amino acid sequence alignment of known *Ganoderma* FIPs. FIP-gap1 (GenBank: AEP68179.1) and FIP-gap2 (GenBank: ART88472.1), FIP-gat (GenBank: AJD79556.1), FIP-gbo (Li et al., 2016), FIP-gja (GenBank: AAX98241.1), FIP-gmi (Lin et al., 2016), FIP-gsi (Li et al., 2011), FIP-gts (Lin et al., 1997), LZ-8 (GenBank: AAA33350.1) and LZ-9 (Bastiaan. et al., 2013) were from *G. applanatum*, *G. atrum*, *G. boninense*, *G. japonicum*, *G. microsporum*, *G. sinense*, *G. tsugae* and *G. lucidum*, respectively. Identical amino acid residues are marked in black shades, whereas similar amino acids are shaded in pink or light blue

**Fig. 2:**
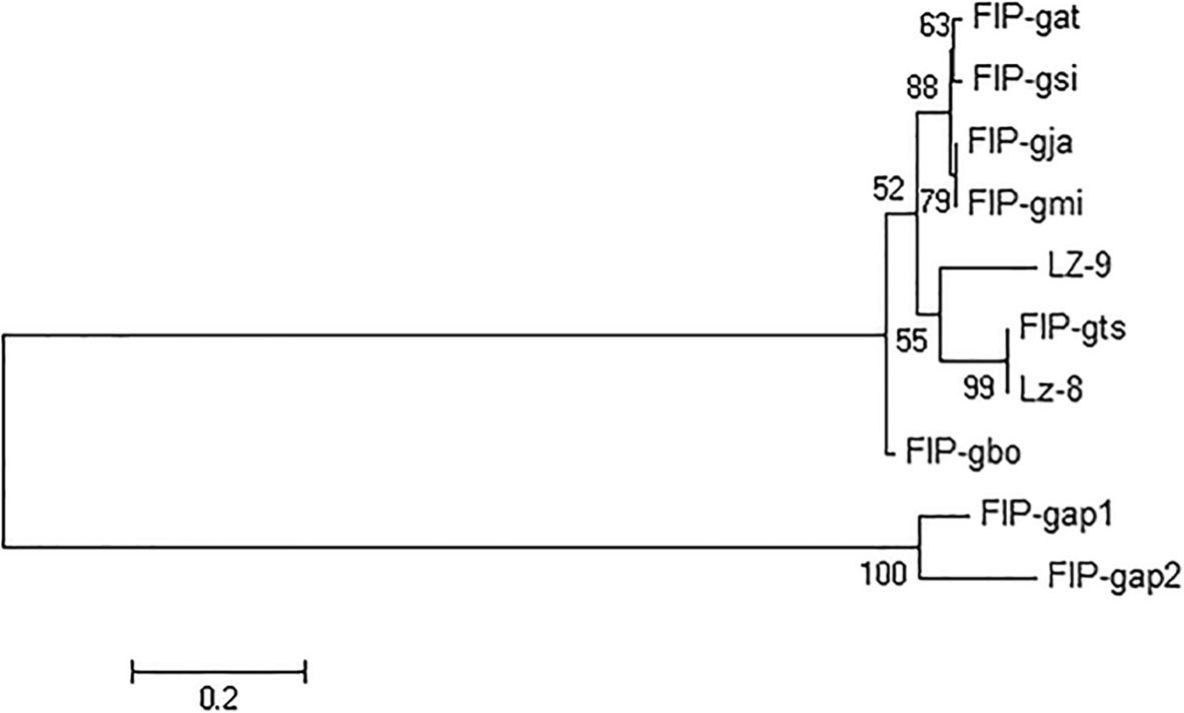
Evolutionary relationship among identified *Ganoderma* FIPs. Phylogenetic tree of *Ganoderma* FIPs constructed by MEGA software (version 7.0) using the neighbour-joining method

FIPs are highly conserved eukaryotic proteins and different FIPs exhibit homology. Hence, homology between *Ganoderma* FIPs was primarily analyzed by amino acid sequence alignment using DNAMAN software. The NCBI’s protein BLAST showed that *Ganoderma* FIPs shared high homology, ranging from 72.6 to 100% (Fig. 1). For example, LZ-8 and FIP-gts, FIP-gja and FIP-gmi were 100% identical. FIP-gsi, FIP-gas, FIP-gja and FIP-gmi were of 98% identity. Furthermore, similarities of FIP-gap1 and FIP-gap2 with LZ-8 were 77.9 and 72.6% in their amino acid sequences. And FIP-gsi shared 78.7 and 76.1% homology with FIP-gap1 and FIP-gap2, respectively (Fig. 1; Additional file 1). Phylogenetic tree of *Ganoderma* FIPs constructed by MEGA (version 7.0) revealed that FIPs from *Ganoderma* species mainly clustered into three lineages (Fig. 2). Firstly, FIP-gat, FIP-gsi, FIP-gja and FIP-gmi formed a big separate lineage. While LZ-8, FIP-gts and LZ-9 clustered into another second lineage. The third lineage included only two FIPs, FIP-gap1 and FIP-gap2, both from *G. applanatum*.

### Gene synthesis and recombinant vector construction

**Fig. 3:**
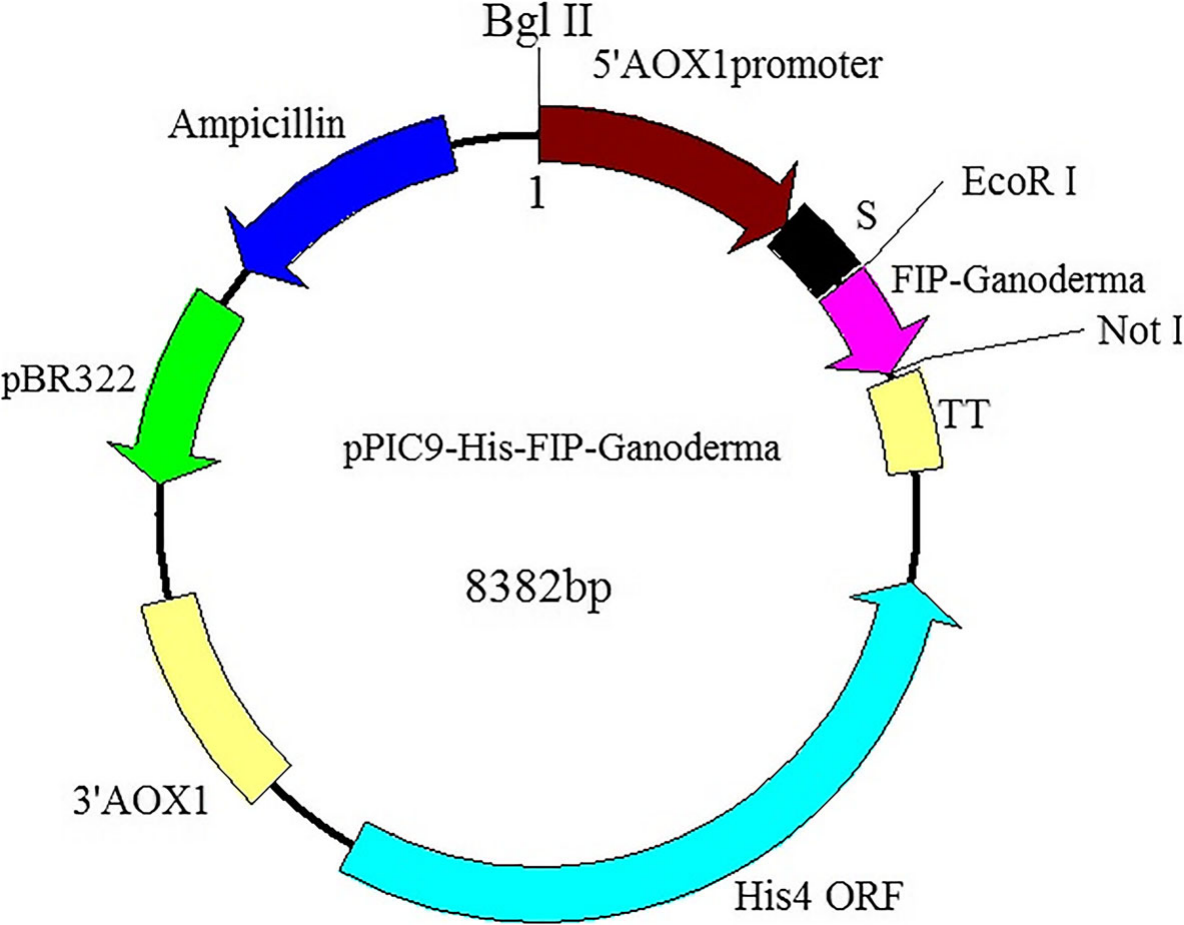
Schematic plasmid map of recombinant expression construct, denoted as pPIC9-His-FIP-Ganoderma. The target *Ganoderma* FIP genes (in pink) were respectively inserted into pPIC9 under the promoter P_AOX_ and α-signal peptide

Four codon-optimized *Ganoderma* FIP genes were artificially synthesized by Sango (Shanghai, China), within which a 6 × His tag sequence was also inserted before the stop codon. Codon adaptation indexes (CAI) for *Ganoderma* FIP genes in *P. pastoris* before and after codon-optimization are shown in Additional file 2. The sketch map of synthetic FIP genes is as shown in Additional file 3. The eukaryotic expression vectors were constructed via digestion and ligation using synthetic gene fragments and plasmid pPIC9, transformation of *E. coli* competent cells, and confirmation by colony PCR and sequencing. Finally, recombinant plasmids were obtained and were universally named as pPIC9-His-FIP-Ganoderma (Fig. 3), in which the four *Ganoderma* FIP genes were integrated under the control of the P_**AOX**_ promoter.

### Expression and purification of *Ganoderma* FIPs in *P. pastoris*

**Fig. 4:**
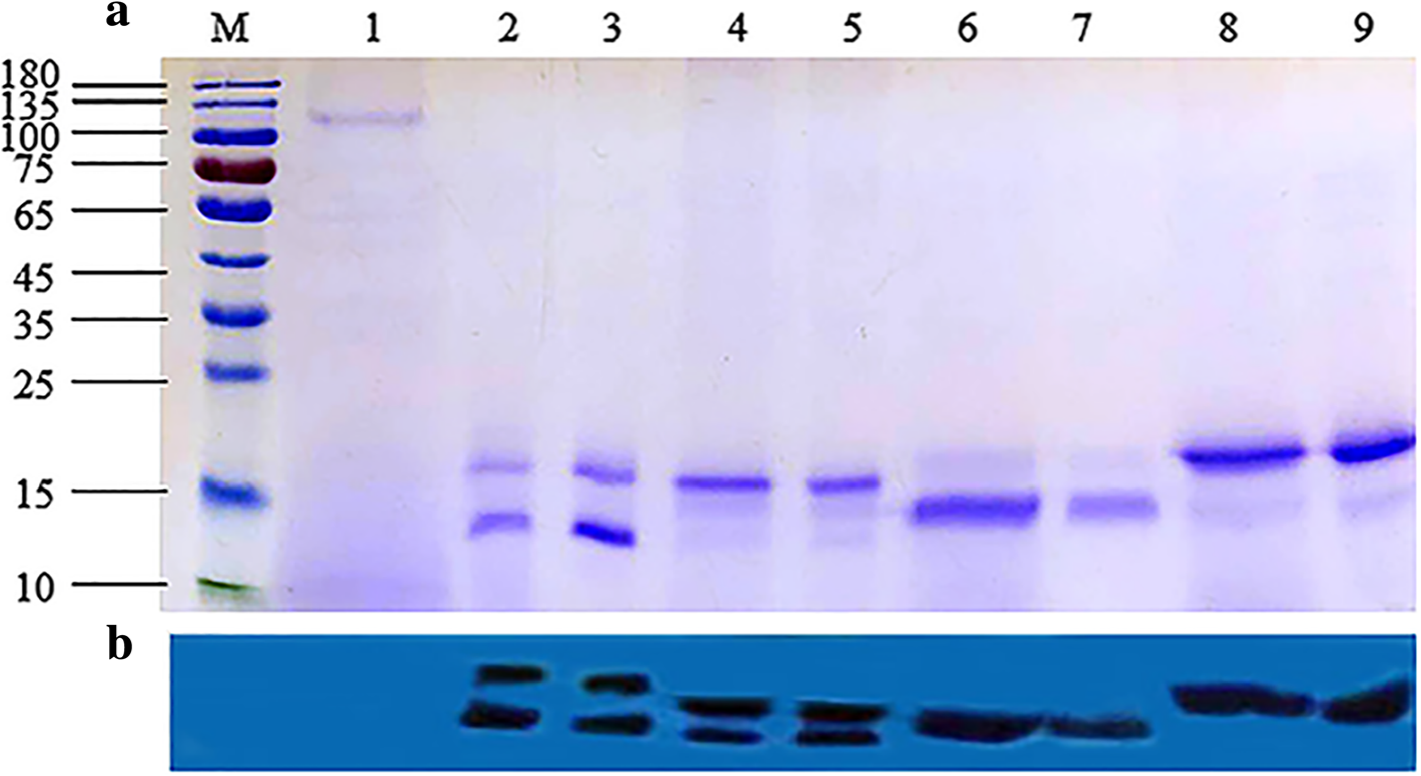
SDS-PAGE (**a**) and Western Blotting (**b**) of the recombinant *Ganoderma* FIPs from *P. pastoris*. Lane M, protein marker; lane 1, supernatant of wild-type GS115, negative control; lane 2 & 3, rFIP-gap1 from fermentation culture and purified rFIP-gap1; lane 4 & 5, cultured and purified rFIP-gap2; lane 6 & 7, rLZ-8 from fermentation culture and purified rLZ-8; lane 8 & 9, rFIP-gsi from cultured supernatant and purified rFIP-gsi

After transformation, the correct positive *P. pastoris* transformants were identified by screening culture on MD (Minimal Dextrose) and colony PCR. The phenotypes of all the yeast transformants were His^+^Mut^s^ after further cultivation on MD and MM (Minimal Methanol) media. Growth and induction culture of yeast transformants were conducted in YPD and BMMY, respectively. Purification of recombinant *Ganoderma* FIPs were performed by one-step chromatography using prepacked HIS Trap™ FF columns. The supernatants from induced culture at 96 h and wild-type yeast, along with the purified *Ganoderma* FIPs, were subjected to SDS-PAGE and Western blot for detection and analyses. Two protein bands of approximately 14 and 17 kDa could be clearly observed for rFIP-gap1 and rFIP-gap2, whereas single protein bands, 14 and 17 kDa, of rLZ-8 and rFIP-gsi were obviously visualized, respectively (Fig. 4a). Moreover, all those protein bands could be obviously immunologically detected by anti-6 × His antibody (Fig. 4b). Besides, the expression level of rFIP-gap1, rFIP-gap2, rLZ-8 and rFIP-gsi were 247.4, 197.5, 253.6 and 264.3 mg L^− 1^ measured by densitometric scanning and quantitative analysis.

### Haemagglutination and mitogenic activities of recombinant *Ganoderma* FIPs

**Fig. 5:**
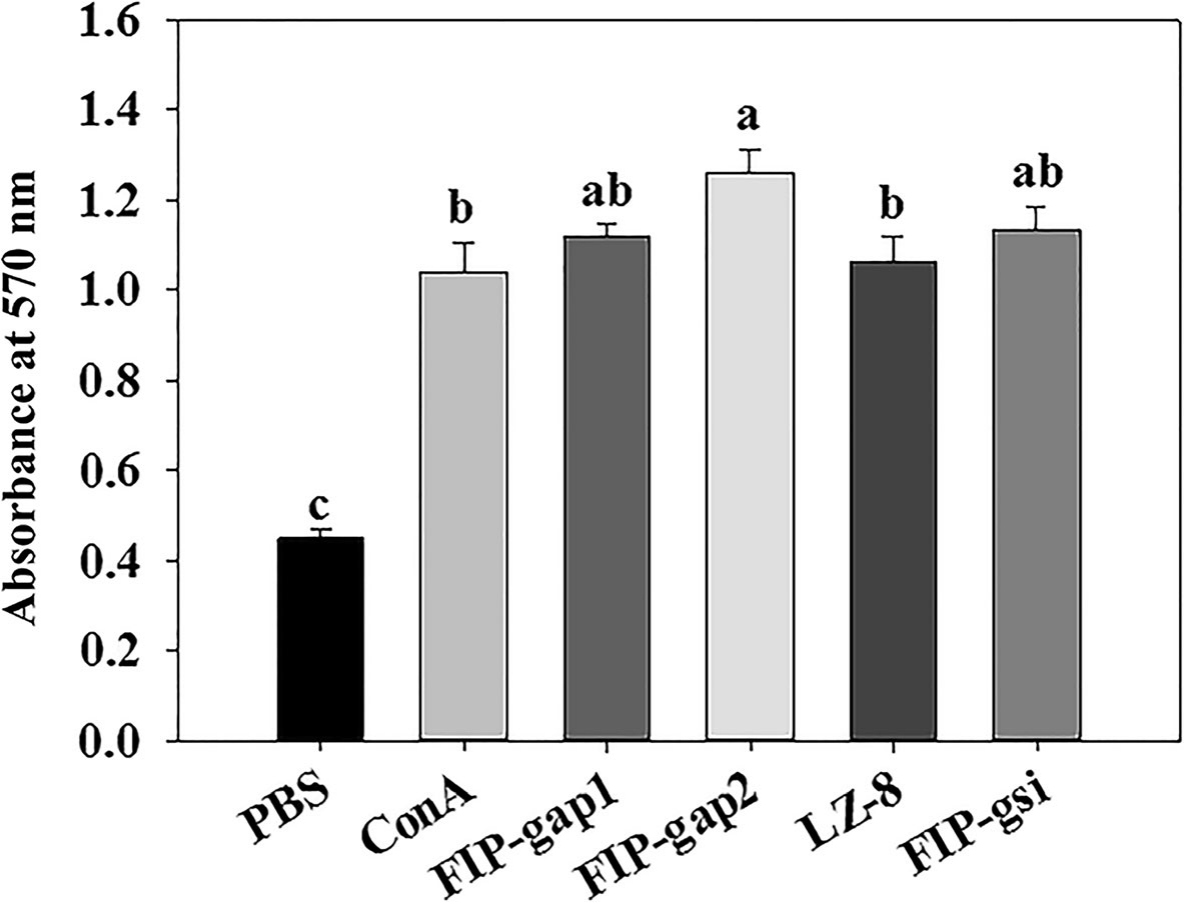
Mitogenesis examination of recombinant *Ganoderma* FIPs on mouse splenocytes using a MTT method. PBS and ConA (5 μg mL^− 1^) were used as negative and positive controls to treat splenocytes. Final concentrations of different rFIPs were 5 μg mL^− 1^, results of which were from triplicate tests. Statistical analysis was performed through one-way ANOVA. Different letters indicate significance (*P* < 0.05) and same letters mean the difference is not significant

Hemagglutination activities of recombinant *Ganoderma* FIPs (all at 5 μg mL^− 1^) towards three different mammalian red blood cells, which was detected using a microscope at magnification of (16 × 20). Results indicated that rFIP-gap1 and rFIP-gsi were able to agglutinate human, sheep and mouse red blood cells, whereas rLZ-8 showed hemagglutination ability to sheep and mouse blood cells, but not the human’s. However, the other FIP from *G. applanatum*, rFIP-gap2, couldn’t agglutinate any of the red blood cells (Table 1; Additional file 4). In addition, PHA (5 μg mL^− 1^) used as a positive control caused haemagglutination towards human, sheep and mouse red blood cells. The MTT method was applied to examine the stimulatory activities of recombinant FIPs, which revealed that all *Ganoderma* FIPs could significantly stimulate the cell viability of murine splenocytes at concentrations of 5 μg mL^− 1^ by contrast with the negative control (Fig. 5). Interestingly, rFIP-gap1, rFIP-gap2 and rFIP-gsi were even more effective in splenocyte mitogenesis than the positive control ConA (5 μg mL^− 1^).

**Table 1:** Haemagglutination test of rFIP-gap1, rFIP-gap2, rLZ-8 and rFIP-gsi

Sources of red blood cells	PBS	PHA	rFIP-gap1	rFIP-gap2	rLZ-8	rFIP-gsi
Human	–	++	+	–	–	++
	–	++	+	–	–	++
Sheep	–	++	++	–	++	++
	–	++	+	–	++	++
Mouse	–	++	++	–	++	++
	–	++	++	–	++	+

Note: Haemagglutinating examination of various *Ganoderma* FIPs including rFIP-gap1, rFIP-gap2, rLZ-8 and rFIP-gsi (all final concentration at 5 μg mL^− 1^) towards human, sheep and mouse red blood cells. PBS and PHA (5 μg mL^− 1^) served as negative and positive controls. (+) indicates positive reaction and (−) indicates negative reaction. (++) and (+) denotes 75 and 50% of cells underwent haemagglutination, respectively. All results were from biological duplicate tests

### Examination of induced cytokines release from murine splenic lymphocytes

**Fig. 6:**
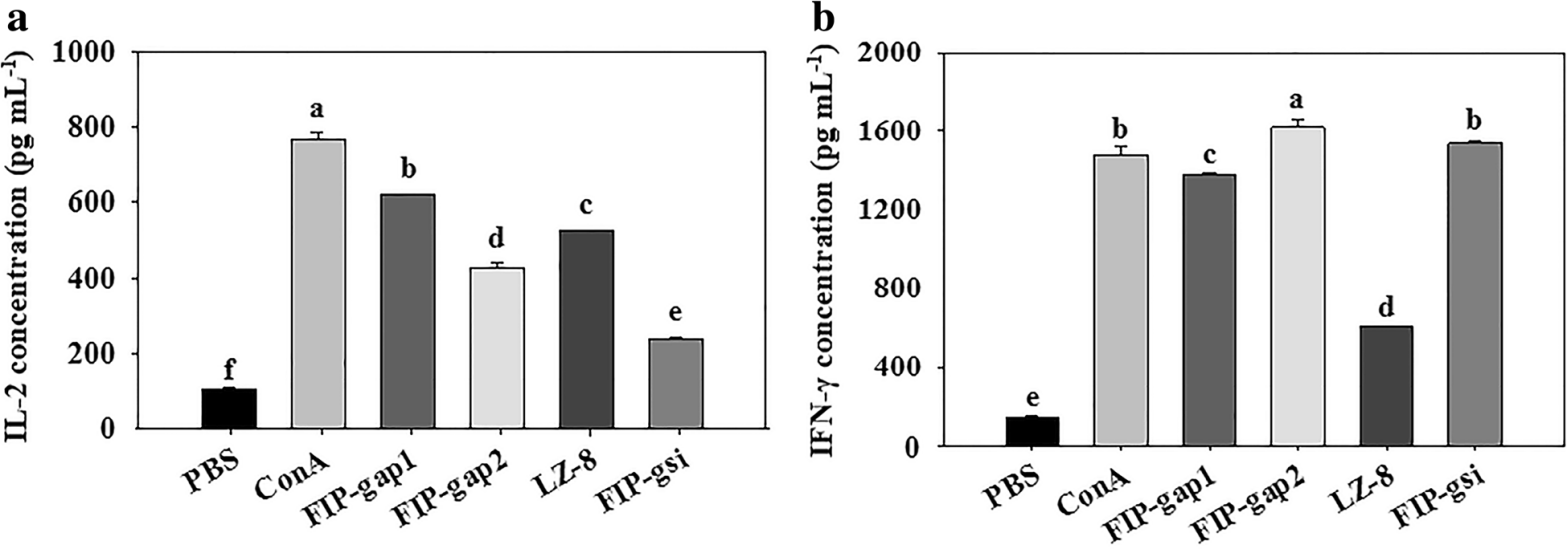
Effect of different *Ganoderma* FIPs on IL-2 (**a**) and IFN-γ (**b**) release from mouse splenocytes. PBS and ConA (5 μg mL^− 1^) served as negative and positive controls. Final concentrations of different rFIPs were 5 μg mL^− 1^. The relative concentrations were calculated from the reading data at 450 nm by ELISA. All data was statistically analyzed through one-way ANOVA. Different letters indicate significance (*P* < 0.05) and same letters mean the difference is not significant

The immunomodulation abilities of *Ganoderma* FIPs were demonstrated by inducing secretion of IL-2 and IFN-γ from murine splenocytes measured via ELISA. Results indicated that compared with the negative control, all the four FIPs (5 μg mL^− 1^) could obviously enhance the expression levels of two cytokines. For IL-2, rFIP-gap1, rFIP-gap2, rLZ-8 and rFIP-gsi increased the release up to 623, 417, 525 and 237 pg mL^− 1^, respectively, while the level of IL-2 secretion induced by ConA (5 μg mL^− 1^), the positive control, reached 769.5 pg mL^− 1^ (Fig. 6a). For IFN-γ, the induced expression levels by rFIP-gap1, rFIP-gap2, rLZ-8 and rFIP-gsi were 1381, 1624, 606 and 1538 pg mL^− 1^, and ConA could increase the level of IFN-γ release to 1483 pg mL^− 1^ (Fig. 6b). Additionally, rFIP-gap2 was even superior to ConA in IFN-γ induction.

### Cytotoxic effect assessment of recombinant *Ganoderma* FIPs

**Fig. 7:**
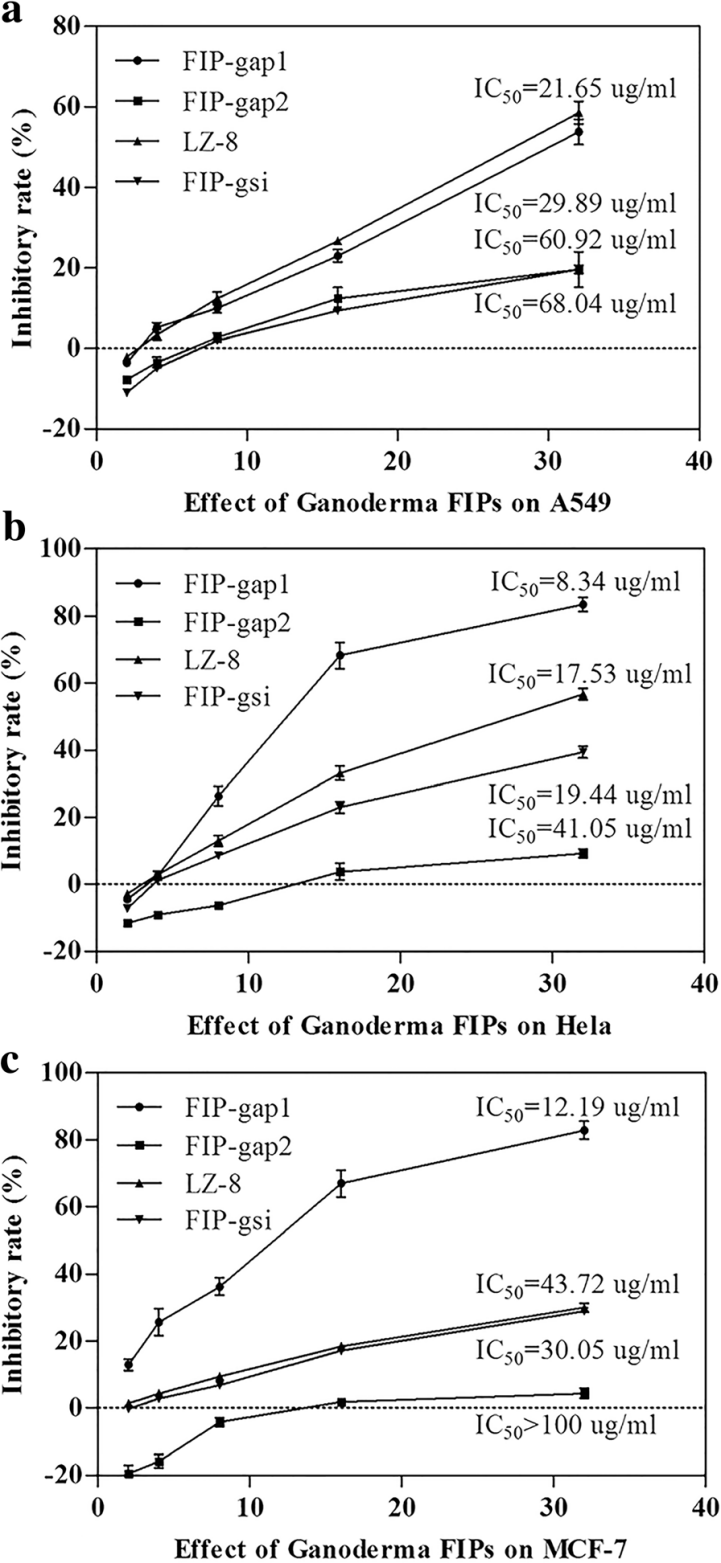
Cytotoxic effects of four *Ganoderma* FIPs towards A549 (**a**), Hela (**b**) and MCF-7 (c) cancer cell lines. All cells were treated with rFIP-gap1, rFIP-gap2, rLZ-8 and rFIP-gsi at concentration ranging from 2 and 32 μg mL^− 1^. The inhibitory rates were calculated according to cell viabilities measured by MTT assays and the half-maximal inhibitory concentration (IC_50_) was deduced from the graphs

The cytotoxic activities of different *Ganoderma* FIPs were demonstrated by their inhibitory effects towards three types of human cancer cells using a MTT method as described above. And different IC_50_ were calculated from the cell viability data using SPSS 19.0 software. Results indicated that rFIP-gap1 showed significant cytotoxic effect towards A549, Hela and MCF-7 cells, with IC_50_ values of 29.89, 8.34 and 12.19 μg/mL, respectively (Fig. 7). rLZ-8 and rFIP-gsi exhibited moderate inhibitory activities to the same cancer cell lines. The IC_50_ values of rLZ-8 were 21.65, 17.53 and 43.72 μg/mL, whereas those of rFIP-gsi were 68.04, 19.44 and 30.05 μg/mL (Fig. 7). rFIP-gap2, the other FIP from *G. applanatum* showed the poorest cytotoxicity. The IC_50_ values of rFIP-gap2 towards A549, Hela and MCF-7 cancer cell lines were 60.92, 41.05 and > 100 μg/mL, respectively (Fig. 7).

## Discussion

*Ganoderma*s have been applied and recorded in China for thousands of years, which are deemed as precious auspicious herbs and thought to prolong life as well as to cure the dying patients [1, 2]. As estimated, there are over 200 *Ganoderma* species worldwide with more than 80 distributed in China, among which *G. lucidum* is the most well-known [3, 39, 40]. Various components from *Ganoderma*s have been proven bioactive including polysaccharides, proteins, glycopeptides, triterpenes etc. Previous studies found that polysaccharides and triterpenes were the two main active substances for *Ganoderma*s [8, 11, 40]. In terms of that, Zhao et al. once stated that *G. sinense* was the most valuable and bio-effective species [40]. However, discovery of FIPs revealed that proteins alone from *Ganoderma*s could exert very similar bioactivities to polysaccharides and triterpenes, such as immunomodulation and anti-tumor activities [23]. Since the first FIP, LZ-8, was identified from *G. lucidum*, seventeen FIPs have been reported to date, among which more than a half are from *Ganoderma*s [24–30]. And these *Ganoderma* FIPs shared very high homology in their amino acid sequences (Fig. 1). On basis of the protein sequence alignment and phylogenetic tree analysis of *Ganoderma* FIPs (Fig. 2), we could divide *Ganoderma*s into three main groups, Lingzhi (*Ganoderma*), Zizhi (*Phaeonema*) and Shushe (*G. applanatum*) in the current paper. That was very similar and accordant with the traditional classification of *Ganoderma*s as well as other sorting results using molecular markers [41]. Therefore, we can speculate that all *Ganoderma*s probably contain FIPs, which can be potentially used as candidate markers for *Ganoderma* classification.

As reported previously, it is time-consuming and costly to extract FIPs from natural mycelia and fruiting bodies with usually low yield [25, 28]. For example, merely 10 mg of LZ-8 was purified from 300 g wet *G. lucidum* mycelia [24]. Hence, researchers have been making efforts in investigating new methods to obtain recombinant FIPs via bio-engineering techniques. For instance, FIP-gts and FIP-fve have been expressed in *E. coli* and insect cells, with yields of ranging from 5 to 20 mg L^− 1^ [28]. Besides, LZ-8 has been expressed in *Bacillus subtilis* and *Lactococcus lactis* at expression levels of 17.4 and 1.24 mg L^− 1^, respectively [42]. However, the expression levels of these rFIPs from prokaryotic and insect cells are commonly very low. Furthermore, recombinant FIPs from *E. coli* possessed only 50% bioactivity of native ones [42]. The *P. pastoris* expression system is regarded as an ideal host and extensively employed to express functional proteins of medicinal or commercial values [28, 31]. Generally, the expression levels are high as a result of the P_**AOX**_ promoter, which is reported to be the strongest promoter to date, and is regulated by methanol [43]. Besides, recombinant heterogeneous proteins can be produced extracellularly and secreted into the culture media with the help of α-signal peptide, which effectively facilitates the downstream processing and purification. In addition, cultivation and fermentation of *P. pastoris* is simple and economical, making it quite applicable for scale-up fermentation and even industrial production [43, 44]. Given all these advantages, *P. pastoris* GS115 was adopted as a host to express the four typical *Ganoderma* FIP genes in the current study. Results exhibited that they were all effectively expressed after codon-optimization, with expression levels of 197.5–264.3 mg L^− 1^ (Fig. 4), which is accordant with previous reports (Table 2) [45]. Additionally, two protein bands of rFIP-gap1 and rFIP-gap2 could be clearly observed in SDSPAGE and Western blotting (Fig. 4). The reason for that was probably due to the post-transcriptional modification including glycosylation, which was a common phenomenon in *P. pastoris* expression system [31, 43]. Potential glycosylation sites were found therein the FIP-gaps’ amino acid sequences, including _38_Asn-_39_Leu-_40_Thr in FIP-gap1 and _31_Asn-_32_Pro-_33_Ser in FIP-gap2.

**Table 2:** Recombinant FIPs produced in *Pichia pastoris*

FIPs	Pichia strains	Vectors	Yields (mg L^− 1^)	Bioactivities	References
LZ-8	GS115	pPIC-9	191.2	hemagglutination, mitogenesis, IL-2 induction, anti-tumor	Lin et al., 2009
	GS115	pPIC-9 K	270	hemagglutination, mitogenesis	Xue et al., 2008
	X-33	pPICZαA	350	not mentioned	Bastiaan et al., 2013
	X-33	pPICZαA	34.4	hemagglutination	Song et al., 2011
	GS115	pPIC-9	253.6	hemagglutination, mitogenesis, IL-2& IFN-γ induction, anti-tumor	Current study
FIP-fve	GS115	pPIC-9	158.2	hemagglutination, mitogenesis, IL-2 & IFN-γ induction	Lin et al., 2013
	X-33	pPICZαA	18.9	hemagglutination	Bastiaan et al., 2013
FIP-vvo	X-33	pPICZαA	410	hemagglutination, mitogenesis, IFN-γ induction	Sun et al.,2014
FIP-nha	X-33	pPICZαA	42.7	hemagglutination	Bastiaan et al., 2013
FIP-cru	GS115	pPIC-9	148.5	hemagglutination, mitogenesis, IL-2 induction	Lin et al., 2016
LZ-9	X-33	pPICZαA	6.7	hemagglutination	Bastiaan et al., 2013
FIP-gsi	GS115	pPIC-9	264.3	hemagglutination, mitogenesis, IL-2& IFN-γ induction, anti-tumor	Current study
FIP-gap1	GS115	pPIC-9	247.4	hemagglutination, mitogenesis, IL-2& IFN-γ induction, anti-tumor	Current study
FIP-gap2	GS115	pPIC-9	197.5	hemagglutination, mitogenesis, IL-2& IFN-γ induction, anti-tumor	Current study

LZ-8, FIP-fve, FIP-vvo, FIP-nha, FIP-cru, LZ-9, FIP-gsi, FIP-gap1 and FIP-gap2 represent FIPs from *G. lucidum*, *Flammulina velutipes*, *Volvariella volvacea*, *Nectria haematococca*, *Chroogomphis rutilus*, *G. lucidum*, *G. sinense* and *G. applanatum*

A series of biological activities have been reported since the discovery of FIPs, including haemagglutination, anti-allergy, immunomodulation, anti-tumor and anti-virus [23–25, 28]. Nevertheless, quite little is known about the similarities and differences between the *Ganoderma* FIPs. Thus, bioactivities were examined and compared specifically between FIP-gap1, FIP-gap2, LZ-8 and FIP-gsi, the four rFIPs from three representative *Ganoderma*s, in terms of haemagglutination, mitogenesis, cytokine induction and cytotoxic effect. Primarily, rFIP-gap1 and rFIP-gsi showed strong haemagglutination ability, as they could agglutinate human, sheep and mouse red blood cells (Table 1). And rLZ-8 was capable to agglutinate sheep and mouse red blood cells but not the human’s, which is identical to natural LZ-8 and rLZ-8 s from *E. coli* or *P. pastoris* [44]. However, rFIP-gap2, the second FIP from *G. applanatum*, could agglutinate none of the three blood cells (Table 1). Later, the immunomodulation activities of these *Ganoderma* FIPs were evaluated by their mitogenesis and cytokine induction towards murine spleen lymphocytes. Results indicated that rFIP-gap2 had the strongest stimulation to enhance cell viability of mouse splenocytes, whereas rFIP-gap1 and rFIP-gsi showed moderate mitogenesis ability, and rLZ-8 was less effective in mitogenic activity (Fig. 5). For cytokine induction test, the ability of *Ganoderma* FIPs to increase IL-2 release were as follows: rFIP-gap1 > rLZ-8 > rFIP-gap2 > rFIP-gsi (Fig. 6a), while the activity of different FIPs to induce IFN-γ expression were: rFIP-gap2 > rFIP-gsi > rFIP-gap1 > rLZ-8 (Fig. 6b). Finally, the cytotoxic effects of different *Ganoderma* FIPs were detected by their inhibition towards different human cancer cells via a MTT method. Results revealed that four *Ganoderma* FIPs showed better cytotoxic activity towards Hela and MCF-7 cancer cells than lung cancer A549. To be more specific, the inhibitory ability of different *Ganoderma* FIPs were: rFIP-gap1 > rLZ-8 > rFIP-gsi > rFIP-gap2, estimated by their IC_50_ towards different cancer cell lines (Fig. 7). Additionally, *Ganoderma* FIPs showed similar but weaker cytotoxic activities by contrast with some other FIPs, for example, FIP-lrh from *Lignosus rhinocerotis*, exhibited stronger cytotoxicity to A549, Hela and MCF-7 cancer cells, with IC_50_ values ranging from 5.07 to 8.94 μg/mL [46]. And the IC_50_ value of another FIP, FIP-sch3 (*Stachybotrys chartarum*), was 10.80 μg/mL [29]. Besides, the cytotoxicity of rFIP-gaps towards A549 and Hela cells in current study was accordant with those in previous reports with very similar IC_50_ values [35].

Conclusively, four FIPs including FIP-gap1, FIP-gap2, LZ-8 and FIP-gsi from three representative *Ganoderma* species were functionally and effectively expressed in *P. pastoris* in present paper, with expression levels of 197.5 to 264.3 mg L^− 1^. And those recombinant *Ganoderma* FIPs possessed ideal bioactivities in vitro. The immunomodulation ability of different *Ganoderma* FIPs was: rFIP-gap2 > rFIP-gap1 > rLZ-8 & rFIP-gsi, whereas the cytotoxicity activity of them was: rFIP-gap1 > rLZ-8 > rFIP-gsi > rFIP-gap2.

## Conclusion

In the current study, a pair of novel FIP genes from *G. applanatum*, along with another two FIP genes from *G. lucidum* and *G. sinense*, were functionally expressed in *P. pastoris* GS115, at yields ranging from 197.5 to 264.3 mg L^− 1^, which were with much higher yield than recombinant FIPs from *E. coli* and insect cells. Bioactivity examination of these four typical *Ganoderma* FIPs indicated that FIP-gap1 and FIP-gap2 showed more immunomodulation activity than LZ-8 and FIP-gsi in terms of mitogenesis and cytokine induction from mouse splenocytes. Besides, cytotoxic effects of these FIPs were: rFIP-gap1 > rLZ-8 > rFIP-gsi > rFIP-gap2 towards three human cancer cell lines after comparison of their IC_50_. Our results showed that similarities and differences indeed existed between different *Ganoderma* FIPs in both their protein sequences and bioactivities. And it seemed that FIPs from *G. applanatum* were more bioactive than FIPs from *G. lucidum* and *G. sinense*. Additionally, our study also confirmed that recombinant FIPs from *P. pastoris* could be used as robust effective resources for further development and investigation.

## Materials and methods

### Strains, plasmids and kits

*P. pastori*s strain GS115 and plasmid pPIC9 were purchased from Invitrogen (USA, Catalog number: K171001). *Escherichia coli* Top10 competent cells were procured from Tiangen (Beijing, China). HisTrap™ FF prepack columns (5 mL) were GE products (USA). Frozen-EZ Yeast Transformation II Kit™ was from Zymo Reasearch (USA). Mouse IL-2 and IFN-gamma ELISA kits were bought from Beyotime (Shanghai, China). Restriction endonucleases, T4 DNA ligase and agarose gel DNA purification kits were Takara (Japan) products. Concanavalin A (ConA) and biotin were from Sigma (USA). Thiazolyl blue and yeast nitrogen base with ammonium sulfate without amino acids (YNB) were purchased from Genview (USA). All the other chemicals were of analytical grade. Gene synthesis and sequencing were carried out by Sangon (Shanghai, China).

### Sequence alignment and phylogenetic tree construction

Up till now, ten *Ganoderma* FIPs have been identified as mentioned above. Primarily, nucleotide and protein sequcences’ BLAST searching and analysing was carried out using NCBI and DNAMAN software (version 8.0). Then the phylogenetic tree of *Ganoderma* FIPs was constructed using the neighbour-joining method by the MEGA in version 7.0 software (http//https://www.megasoftware.net). The statistical confidence in the phylogenetic relationships was assessed with bootstrap tests, which were replicated 1000 times.

### Gene synthesis and vector construction

Four typical FIPs from three famous representative *Ganoderma* species were chosen for targets including LZ-8, FIP-gsi, FIP-gap1 and FIP-gap2. Firstly, the low-usage and rare codons in the original four FIP genes were replaced by high-usage codons, according to the codon bias in *P. pastoris* [47]. Secondly, EcoRI and NotI recognition sequences were added before the start codons and after the stop codons, respectively. In addition, 6 × His-tag sequences were also inserted before stop codons in the C terminal. Then the four optimized *Ganoderma* FIP genes were sent to Sangon for synthesis. Both the synthetic FIP genes and plasmid pPIC9 were digested with EcoRI and NotI at 37 °C for 1.5 h. Then a rapid ligation using target genes and digested pPIC9 was conducted at 22 °C for a half hour. The ligated product was used to transform *E. coli* TOP10 competent cells, which were later screened on LB media with ampicillin (100 μg mL^− 1^). The positive *E. coli* transformants were detected by colony PCR and endonuclease digestion. Finally, the correct recombinant plasmids were obtained and designated as pPIC9-His-FIP-Ganoderma, which were further confirmed by sequencing.

### Transformation and induced expression of *Ganoderma* FIPs

Preparation and transformation of yeast competent cells were performed according to the kit instruction (Catalog number: T2001). Phenotype of yeast transformants was determined by screening on MD and MM media. Positive transformants were verified by colony PCR and cultured in YPD at 30 °C, 200 rpm until OD_600_ ≥ 2.0. Then the yeast cells were harvested by centrifugation at 6000 rpm for 5 min and then resuspended in BMMY 200 mL 500 mL^− 1^ in one flask) to induce protein expression. Methanol was added every 24 h to maintain a final concentration of 1% (*v*/v). At 96 h, 1 mL of the expression cultures were transferred for later use. The wild-type yeast was used as a negative control and cultured in a same way. Purification of recombinant *Ganoderma* FIPs was carried out using HisTrap™ FF prepack columns according to the manufacturer’s protocol (Catalog number: 17–5319-01). The protein masses of recombinant proteins were determined through SDS–PAGE and quantitative analyses with an image-analyzing system (QUANTITY ONE, Bio-Rad, USA) [28].

### SDS-PAGE and Western blot analyses

25 μL supernatants of four recombinant *Ganoderma* FIPs (at induction time of 96 h) and 10 μL purified protein samples were mixed with equal volumes of 2 × loading buffer for SDS-PAGE test. The wild-type yeast supernatant was as a negative control. All the mixtures were boiled for 5 min before electrophoresis. SDS-PAGE was performed in 12% acrylamide gels and visualized by staining with Coomassie brilliant blue R250. For Western blot, after SDS–PAGE, proteins samples were transferred onto a PVDF membrane. After blocking with 5% nonfat dry milk in TBST and incubated with anti-His monoclonal antibody raised in mouse (Tiangen, China, 1:25000 diluted) for two hours. The membrane was washed five times with TBST and incubated with horseradish peroxidase (HRP)-conjugated anti-mouse IgG raised in goat (Tiangen, 1:5000 diluted) for 1.5 h at 37 °C [43].After incubation with the ECL reagent (Pierce, USA), the membrane was subjected to X-ray film for exposure 2–5 s at totally dark conditions. Subsequently, the X-ray film was developed in the developer bath for 15–20 s, and then fixed in the fixer solution.

### Haemagglutination and mitogenesis assays of recombinant FIPs

After purification, the agglutination activity of recombinant *Ganoderma* FIPs was primarily tested. 50 μL of purified rFIP-gap1, rFIP-gap2, rLZ-8 and rFIP-gsi (final concentration of 5 μg mL^− 1^) in PBS were mixed with equal volumes of human, sheep and mouse red blood cells (1.5%, *v*/v) in a 96-well microplate, respectively. PHA (5 μg mL^− 1^) served as a positive control, whereas PBS was used as a negative control. The plate was incubated in a 5% CO_2_ incubator at 37 °C after gently mixed and haemagglutination was examined after 1.5 and 24 h. The mitogenic activity of *Ganoderma* FIPs was demonstrated by their stimulatory effect towards mouse splenocytes. Homogenous splenocytes of male Balb/c mice (4–6 weeks old, purchased from the Pharmacology Experimental Center of Shenyang Agriculture University) were grown and suspended to 1 × 10^6^ cells mL^− 1^ in RPMI 1640 medium. Subsequently, 100 μL cell suspension and recombinant *Ganoderma* FIPs (5 μg mL^− 1^) were seeded into a 96-well plate. Concanavalin A (ConA, 5 μg mL^− 1^) and PBS served as positive and negative controls. The cell viability was evaluated by a MTT method. In brief, after the cells were cultured in 5% CO_2_ for 48 h at 37 °C, 20 μL MTT (5 μg mL^− 1^) was added, and the cell mixtures were incubated for another 4 h. The cell supernatants were carefully removed and 100 μL dimethyl sulfoxide (DMSO) was added into the plate. The plate was then read on a microplate reader (Model 680, Bio-Rad) at 570 nm. The experimental procedures have been approved by the Ethics Committee for Laboratory Animal Care (Animal Ethics Procedures and Guidelines of the People’s Republic of China) for the use of Shenyang Agricultural University, China. (Permit No. 264 SYXK<Liao> 2011–0001).

### Cytokines induction detection

To test the immunomodulatory bioactivities of recombinant *Ganoderma* FIPs, the expression levels of induced IL-2 and IFN-γ released from the murine splenocytes were detected by ELISA method. The murine splenocytes were prepared as above and adjusted to 1 × 10^7^ cells mL^− 1^ in RPMI 1640 medium. Then 100 μL cells plus equal volumes of rFIP-gap1, rFIP-gap2, rLZ-8 and rFIP-gsi (final concentration of 5 μg mL^− 1^) were mixed and seeded into a 96-well plate. Meanwhile, PBS and ConA (5 μg mL^− 1^) were used as controls. After the plate was cultured in 5% CO_2_ at 37 °C for 48 h, the supernatants of the cells were collected. Expression levels of mouse IL-2 and IFN**-**γ in the supernatants were measured using ELISA kits (Multi Sciences, Hangzhou, China) in accordance with the manufacturer’s instructions, and serial concentrations (7.81, 15.63, 31.2, 62.5, 125 and 250 pg mL^− 1^) and (62.50, 125, 250, 500, 1000 and 200 pg mL^− 1^) of mouse IL-2 and IFN-γ were prepared as standards, respectively [28, 43].

### Cytotoxicity examination of recombinant *Ganoderma* FIPs

To determine cytotoxic activities, three cancer cell lines, human lung carcinoma A549, cervical carcinoma Hela and human breast carcinoma MCF-7 were used for test, which were provided by the Cell Research Center, College of Veterinary Science and Animal Husbandry (SYAU). Those cancer cells were cultured in DMEM (Tiangen) supplemented with 10% FBS, 100 units/mL penicillin and 100 μg mL^− 1^streptomycin at 37 °C in a 5% CO_2_ incubator. Subsequently, the cells were trypsinized with 0.25% trypsin containing 0.04% EDTA and adjusted to 5 × 10^5^ cells mL^− 1^. 100 μL cancer cells were seeded in triplicate wells with diluted rFIP-gap1, rFIP-gap2, rLZ-8 and rFIP-gsi (final concentration of 2, 4, 8, 16 and 32 μg mL^− 1^), which were continually cultured for another 24 h. Meanwhile, PBS was added into these cell cultures in a same way as a negative control. Inhibition of cancer cell growth was presented using the half-maximal inhibitory concentration (IC_50_), which was defined as the concentration causing 50% inhibition of cell proliferation [28].

### Data analysis

Data were presented as the mean ± SD of three separate experiments performed in duplicate. Statistical analysis was performed through means of one-way ANOVA and the LSD-test with SPSS 19.0 software. Differences were considered to be statistically significant when the *P* < 0.05.

## Additional files

Additional file 1: Homology between different *Ganoderma* FIPs based on their peptide sequence alignment using a DNAman software (Version 8.0). FIP-gap1 and FIP-gap2, FIP-gat, FIP-gbo, FIP-gja, FIP-gmi, FIP-gsi, FIP-gts, LZ-8 and LZ-9 represented FIPs from *G. applanatum*, *G. atrum*, *G. boninense*, *G. japonicum*, *G. microsporum*, *G. sinense*, *G. tsugae* and *G. lucidum*, respectively. (DOCX 411 kb)

Additional file 2: Codon adaptation indexes (CAI) for *Ganoderma* FIP genes in *P. pastoris*. *FIP-gap1*, *FIP-gap2*, *LZ-8*, and *FIP-gsi* represent FIP genes from *G. applanatum*, *G. lucidum* and *G. sinense*. (DOCX 13 kb)

Additional file 3: Schematic map of synthetic *Ganoderma* FIP genes. Four codon-optimized FIP genes were synthesized (in green) by Sango (Shanghai, China), in which His-tag sequences (in yellow) were also inserted before stop codons. (DOCX 74 kb)

Additional file 4: Haemagglutination examination of four recombinant *Ganoderma* FIPs including rFIP-gap1, rFIP-gap2, rLZ-8 and rFIP-gsi (all final concentration at 5 μg mL^− 1^) towards human (hRBCs), sheep (sRBCs) and mouse (mRBCs) red blood cells, respectively. PBS and PHA (5 μg mL^− 1^) served as negative and positive controls. All results were from biological duplicate tests. (DOCX 1042 kb)

## Abbreviations

ConA: Concanavalin A
DMSO: Dimethyl sulfoxide
FIP: Fungal immunomodulatory protein
HRP: Horseradish peroxidase
IFN-γ: Interferon gamma
IL-2: Interleukin-2
PCR: Polymerase chain reaction
PHA: phytohaemagglutinin
SDS-PAGE: Sodium dodecyl sulphate-polyacrylamide gel electrophoresis

## References

[1] Mao XL (2000). The Macrofungi in China.

[2] Xu JT (1997). Chinese medical mycology.

[3] Chang S, Buswell JA (1999). *Ganoderma lucidum* (Curt.: Fr.) P. karst. (*Aphyllophoromycetideae*)−a mushrooming medicinal mushroom. Int J Med Mushrooms.

[4] Wang YY, Khoo KH, Chen ST, Lin CC, Wong CH, Lin CH (2002). Studies on the immuno-modulating and antitumor activities of *Ganoderma lucidum* (Reishi) polysaccharides: functional and proteomic analyses of a fucose-containing glycoprotein fraction responsible for the activities. Bioorg Med Chem.

[5] Lin ZB (2005). Cellular and molecular mechanisms of immuno-modulation by *Ganoderma lucidum*. J Pharmacol Sci.

[6] Zhang W, Tao J, Yang X, Yang Z, Zhang L, Liu H, Wu K, Wu J (2014). Antiviral effects of two *Ganoderma lucidum* triterpenoids against enterovirus 71 infection. Biochem Biophys Res Commun.

[7] Ergun B (2017). Evaluation of antimicrobial, cytotoxic and genotoxic activities of *Ganoderma lucidum* (Reishi mushroom). Pak J Pharm Sci.

[8] Ferreira IC, Heleno SA, Reis FS, Stojkovic D, Queiroz MJ, Vasconcelos MH, Sokovic M (2015). Chemical features of *Ganoderma* polysaccharides with antioxidant, antitumor and antimicrobial activities. Phytochemistry.

[9] Chang UM, Li CH, Lin LI, Huang CP, Kan LS, Lin SB (2006). Ganoderiol F. a *Ganoderma* triterpene, induces senescence in hepatoma HepG2 cells. Life Sci.

[10] Ngai PH, Ng TB (2004). A mushroom (*Ganoderma capense*) lectin with spectacular thermostability, potent mitogenic activity on splenocytes, and antiproliferative activity toward tumor cells. Biochem Biophys Res Commun.

[11] Jiang Y, Chang Y, Liu Y, Zhang M, Luo H, Hao C, Zeng P, Sun Y, Wang H, Zhang L (2017). Overview of *Ganoderma sinense* polysaccharide-an adjunctive drug used during concurrent chemo/radiation therapy for cancer treatment in China. Biomed Pharmacother.

[12] Gill BS, Navgeet Mehra R, Kumar V, Kumar S (2018). Ganoderic acid, lanostanoid triterpene: a key player in apoptosis. Investig New Drugs.

[13] Smania EF, Delle Monache F, Smania A, Yunes RA, Cuneo RS (2003). Antifungal activity of sterols and triterpenes isolated from *Ganoderma annulare*. Fitoterapia.

[14] Yang G, Yang L, Zhuang Y, Qian X, Shen Y (2016). *Ganoderma lucidum* polysaccharide exerts anti-tumor activity via MAPK pathways in HL-60 acute leukemia cells. J ecept Signal Transduct Res.

[15] Meng LZ, Xie J, Lv GP, Hu DJ, Zhao J, Duan JA, Li SP (2014). A comparative study immunomodulatory activity of polysaccharides from two official species of *Ganoderma* (Lingzhi). Nutr Cancer.

[16] Lv X, Chen D, Yang L, Zhu N, Li J, Zhao J, Hu Z, Wang FL, Zhang LW (2016). Comparative studies on the immunoregulatory effects of three polysaccharides using high content imaging system. Int J Biol Macromol.

[17] Huang S, Mao J, Ding K, Zhou Y, Zeng X, Yang W, Wang P, Zhao C, Yao J, Xia P, Pei G (2017). Polysaccharides from *Ganoderma lucidum* promote cognitive function and neural progenitor proliferation in mouse model of Alzheimer's disease. Stem Cell Reports.

[18] Huang SZ, Ma QY, Kong FD, Guo ZK, Cai CH, Hu LL, Zhou LM, Wang Q, Dai HF, Mei WL, Zhao YX (2017). Lanostane-type triterpenoids from the fruiting body of *Ganoderma calidophilum*. Phytochemistry.

[19] Wang H, Ng TB (2006). Ganodermin, an antifungal protein from fruiting bodies of the medicinal mushroom *Ganoderma lucidum*. Peptides.

[20] Du M, Wang C, Hu XS, Zhao GH (2008). Biological properties of different protein extracts from selenium-enriched *Ganoderma lucidum*. Int J Food Sci Nutr.

[21] Xu XF, Yan HD, Chen J, Zhang XW (2011). Bioactive proteins from mushrooms. Biotechnol Advances.

[22] Wong KL, Wong RN, Zhang L, Liu WK, Ng TB, Shaw PC, Kwok PC, Lai YM, Zhang ZJ, Zhang Y, Tong Y, Cheung HP, Lu J, Sze SC (2014). Bioactive proteins and peptides isolated from Chinese medicines with pharmaceutical potential. Chin Med.

[23] Li QZ, Wang XF, Zhou XW (2011). Recent status and prospects of the fungal immunomodulatory protein family. Crit Rev Biotechnol.

[24] Kino K, Yamashita A, Yamaoka K, Watanabe J, Tanaka S, Ko K, Shimizu K, Tsunoo H (1989). Isolation and characterization of a new immunomodulatory protein, Lingzhi-8 (LZ-8), from *Ganoderma lucidum*. J Biol Chem.

[25] Ko JL, Hsu CI, Lin RH, Jai CL, Lin JY (1995). A new fungal immunomodulatory protein, FIP-fve isolated from the edible mushroom, *Flammulina velutipes* and its complete amino acid sequence. Eur J Biochem.

[26] Lin WH, Huang CH, Hsu CI, Lin JY (1997). Dimerization of the N-terminal amphipathic-helix domain of the fungal immunomodulatory protein from *Ganoderma tsugae* (Fip-gts) defined by a yeast two-hybrid system and site- directed mutagenesis. J Biol Chem.

[27] Li SY, Shi LJ, Ding Y, Nie Y, Tang XM (2015). Identification and functional characterization of a novel fungal immunomodulatory protein from Postia placenta. Food Chem Toxicol.

[28] Lin JW, Guan SY, Duan ZW, Shen YH, Fan WL, Chen LJ, Zhang L, Li TL (2016). Gene cloning of a novel fungal immunomodulatory protein from *Chroogomphis rutilus* and its expression in *Pichia pastoris*. J Chem Technol Biotechnol.

[29] Li S, Zhao L, Xu W, Jiang Z, Kang J, Wang F, Xin F (2016). Identification and characterisation of a novel protein FIP-sch3 from *Stachybotrys chartarum*. PLoS One.

[30] Li S, Jiang Z, Sun L, Liu X, Huang Y, Wang F, Xin F (2017). Characterization of a new fungal immunomodulatory protein, FIP-dsq2 from *Dichomitus squalens*. J Biotechnol.

[31] Bastiaan-Net S, Chanput W, Hertz A, Zwittink RD, Mes JJ, Wichers HJ (2013). Biochemical and functional characterization of recombinant fungal immunomodulatory proteins (rFIPs). Int Immunopharmacol.

[32] Li QZ, Wang XF, Chen YY, Lin J, Zhou XW (2010). Cytokines expression induced by *Ganoderma sinensis* fungal immunomodulatory proteins (FIP-gsi) in mouse spleen cells. Appl Biochem Biotechnol.

[33] Xu H, Kong YY, Chen X, Guo MY, Bai XH, Lu YJ, Li W, Zhou XW (2016). Recombinant FIP-gat, a fungal immunomodulatory protein from *Ganoderma atrum*, induces growth inhibition and cell death in breast cancer cells. J Agric Food Chem.

[34] Lin JW, Duan ZW, Guan SY, Han X, Fan WL, Li HG, Zhang L, Chen SS, Li TL, Cloning G (2016). Bioinformatic analysis and eukaryotic expression vector construction of FIP-gap Fene from *Ganoderma applanatum*. Journal of Shenyang Agricultural University.

[35] Zhou SY, Guan SX, Duan ZW, Han X, Zhang X, Fan WL, Li HG, Chen LJ, Ma H, Liu HM, Ruan YY, Lin JW (2018). Molecular cloning, codon-optimized gene expression and bioactivity assessment of two novel fungal immunomodulatory proteins from *Ganoderma applanatum* in *Pichia*. Appl Microbiol Biotechnol.

[36] Lee YT, Wu CT, Sun HL, Ko JL, Lue KH (2017). Fungal immunomodulatory protein-fve could modulate airway remodel through by affect IL17 cytokine. J Microbiol Immunol Infect.

[37] Wang TY, Yu CC, Hsieh PL, Liao YW, Yu CH, Chou MY (2017). GMI ablates cancer stemness and cisplatin resistance in oral carcinomas stem cells through IL-6/Stat3 signalling inhibition. Oncotarget.

[38] Zhang XQ, Zhao JD (2000). Chinese Flora, Beijing, volume 18.

[39] Zhao JD, Xu LW, Zhang XQ (1984). Studies on the taxonomy of Ganodermataceae in China V. Mycosystema.

[40] Zhao JD, Zhang XQ (1992). Resources and distribution of Ganodermataceae in China. Mycosystema.

[41] Su CL, Tang CH, Zhang JS, Pan YJ (2006). The genetic relationships of isolates of *Ganoderma* inferred from partial β-tubulin gene sequences. Mycosystema.

[42] Yeh CM, Yeh CK, Hsu XY, Luo QM, Lin MY (2008). Extracellular expression of a functional recombinant *Ganoderma lucidum* immunomodulatory protein by *Bacillus subtilis* and *Lactococcus lactis*. Appl Environ Microbiol.

[43] Lin JW, Jia J, Shen YH, Zhong M, Chen LJ, Li HG, Ma H, Guo ZF, Qi MF, Liu LX, Li TL (2013). Functional expression of FIP-fve, a fungal immunomodulatory protein from the edible mushroom *Flammulina velutipes* in *Pichia pastoris* GS115. J Biotechnol.

[44] Lin JW, Hao LX, Xu GX, Sun F, Gao F, Zhang R, Liu LX (2009). Molecular cloning and recombinant expression of a gene encoding a fungal immunomodulatory protein from *Ganoderma lucidum* in *Pichia pastoris*. World J Microbiol Biotechnol.

[45] Song XZ, Xiao JY, Gong BL, Xi ML, Li G (2011). Highly efficient expression of a kind of immunregulator (LZ-8) from *Gannoderma lucidum* in *Pichia pastoris*. Progress in Microbiology and Immunology.

[46] Pushparajah V, Fatima A, Chong CH, Gambule TZ, Chan CJ, Ng ST, Tan CS, Fung SY, Lee SS, Tan NH, Lim RL (2016). Characterisation of a new fungal immunomodulatory protein from Tiger Milk mushroom *Lignosus rhinocerotis*. Sci Rep.

[47] Zhao X, Huo KK, Li YY (2000). Synonymous codon usage in *Pichia pastoris*. Chinese J Biotechnol.

