# Supplementary material for: Recombinant Expression and Bioactivity Comparison of Four Typical Fungal Immunomodulatory Proteins from Three Main Ganoderma Species
Source: BMC Biotechnol. 2018 Dec 14;18:80. doi: 10.1186/s12896-018-0488-0 (PMC6295072; doi:10.1186/s12896-018-0488-0)

**Additional file 1**: Homology between different *Ganoderma* FIPs based on their peptide sequence alignment using a DNAman software (Version 8.0). FIP-gap1 and FIP-gap2, FIP-gat, FIP-gbo, FIP-gja, FIP-gmi, FIP-gsi, FIP-gts, LZ-8 and LZ-9 represented FIPs from *G. applanatum*, *G. atrum*, *G. boninense*, *G. japonicum*, *G. microsporum*, *G. sinense*, *G. tsugae* and *G. lucidum*, respectively.


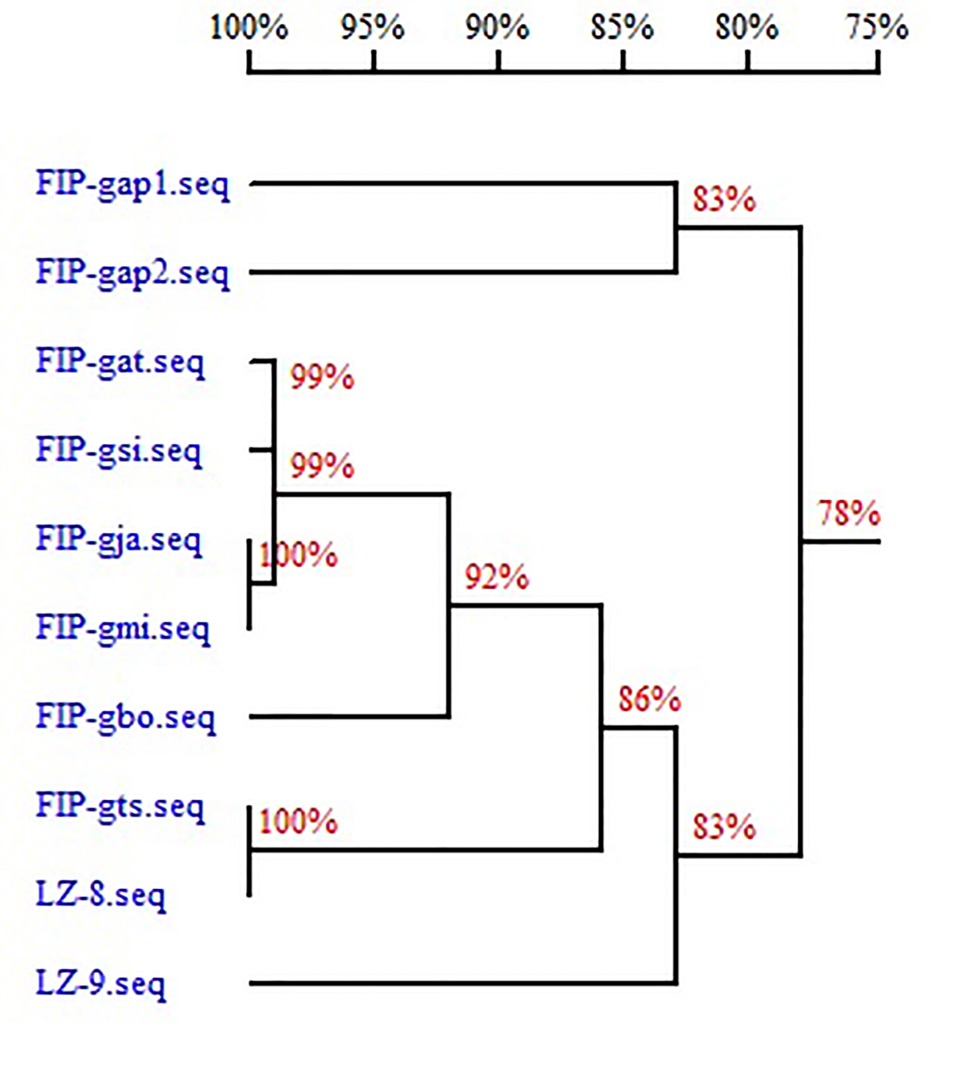

Supplement: Supplementary file 1 — Homology between different Ganoderma FIPs based on their peptide sequence alignment using a DNAman software (Version 8.0). FIP-gap1 and FIP-gap2, FIP-gat, FIP-gbo, FIP-gja, FIP-gmi, FIP-gsi, FIP-gts, LZ-8 and LZ-9 represented FIPs from G. applanatum, G. atrum, G. boninense, G. japonicum, G. microsporum, G. sinense, G. tsugae and G. lucidum, respectively. (DOCX 411 kb) [file 12896_2018_488_MOESM1_ESM.docx]
