# Supplementary material for: Recombinant Expression and Bioactivity Comparison of Four Typical Fungal Immunomodulatory Proteins from Three Main Ganoderma Species
Source: BMC Biotechnol. 2018 Dec 14;18:80. doi: 10.1186/s12896-018-0488-0 (PMC6295072; doi:10.1186/s12896-018-0488-0)
Supplement: Supplementary file 2 — Codon adaptation indexes (CAI) for Ganoderma FIP genes in P. pastoris. FIP-gap1, FIP-gap2, LZ-8, and FIP-gsi represent FIP genes from G. applanatum, G. lucidum and G. sinense. (DOCX 13 kb) [file 12896_2018_488_MOESM2_ESM.docx]

**Additional file 2**: Codon adaptation indexes (CAI) for *Ganoderma* FIP genes in *P. pastoris*. *FIP-gap1*, *FIP-gap2*, *LZ-8*, and *FIP-gsi* represent FIP genes from *G. applanatum*, *G. lucidum* and *G. sinense*.

Table 1S Codon adaptation indexes (CAI) for *Ganoderma* FIP genes in *P. pastoris*

| FIP genes | CAI (before codon-optimization) | CAI (after codon-optimization) |
| --- | --- | --- |
| *FIP-gap1* | 0.53 | 0.83 |
| *FIP-gap2* | 0.55 | 0.83 |
| *LZ-8* | 0.56 | 0.87 |
| *FIP-gsi* | 0.58 | 0.86 |
