# Supplementary material for: Recombinant Expression and Bioactivity Comparison of Four Typical Fungal Immunomodulatory Proteins from Three Main Ganoderma Species
Source: BMC Biotechnol. 2018 Dec 14;18:80. doi: 10.1186/s12896-018-0488-0 (PMC6295072; doi:10.1186/s12896-018-0488-0)

**Additional file 3**: Schematic map of synthetic *Ganoderma* FIP genes. Four codon-optimized FIP genes were synthesized (in green) by Sango (Shanghai, China), in which His-tag sequences (in yellow) were also inserted before stop codons.


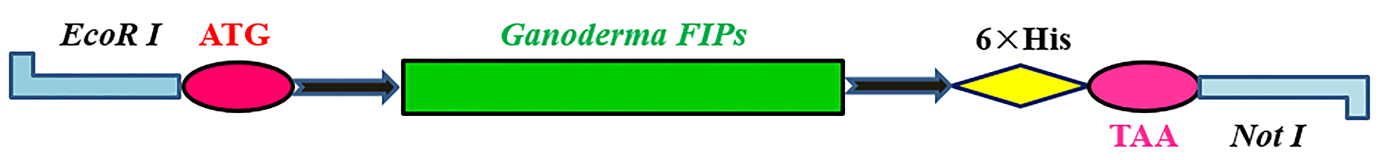

Supplement: Supplementary file 3 — Schematic map of synthetic Ganoderma FIP genes. Four codon-optimized FIP genes were synthesized (in green) by Sango (Shanghai, China), in which His-tag sequences (in yellow) were also inserted before stop codons. (DOCX 74 kb) [file 12896_2018_488_MOESM3_ESM.docx]
