# Supplementary material for: Recombinant Expression and Bioactivity Comparison of Four Typical Fungal Immunomodulatory Proteins from Three Main Ganoderma Species
Source: BMC Biotechnol. 2018 Dec 14;18:80. doi: 10.1186/s12896-018-0488-0 (PMC6295072; doi:10.1186/s12896-018-0488-0)

**Additional file 4**: Haemagglutination examination of four recombinant *Ganoderma* FIPs including rFIP-gap1, rFIP-gap2, rLZ-8 and rFIP-gsi (all final concentration at 5 μg mL^-1^) towards human (hRBCs), sheep (sRBCs) and mouse (mRBCs) red blood cells, respectively. PBS and PHA (5 μg mL^-1^) served as negative and positive controls. All results were from biological duplicate tests.


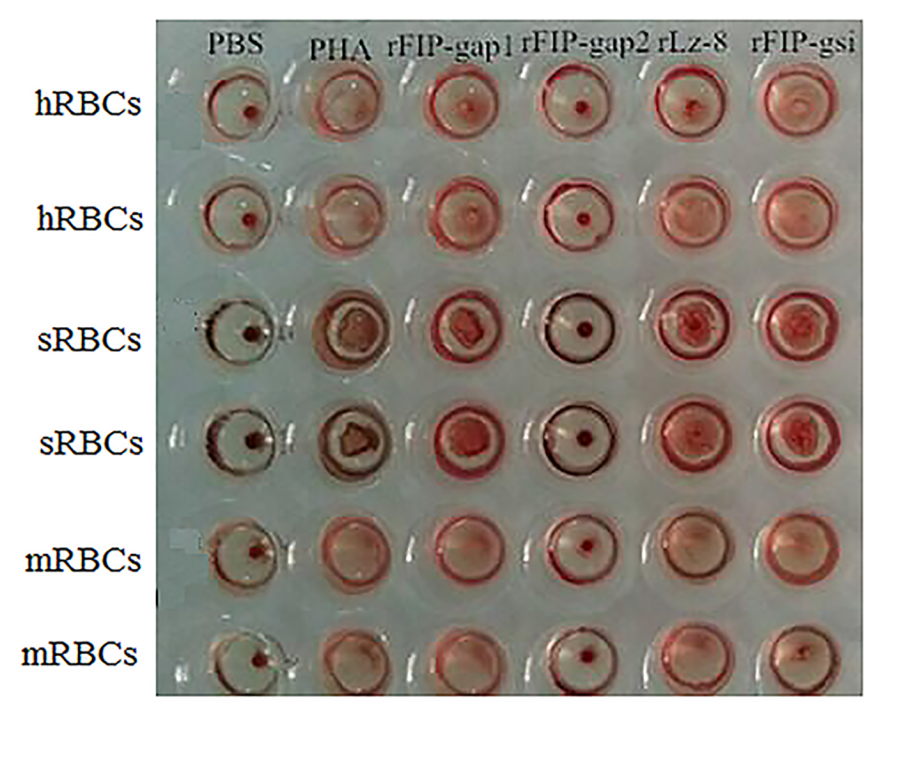

Supplement: Supplementary file 4 — Haemagglutination examination of four recombinant Ganoderma FIPs including rFIP-gap1, rFIP-gap2, rLZ-8 and rFIP-gsi (all final concentration at 5 μg mL− 1) towards human (hRBCs), sheep (sRBCs) and mouse (mRBCs) red blood cells, respectively. PBS and PHA (5 μg mL− 1) served as negative and positive controls. All results were from biological duplicate tests. (DOCX 1042 kb) [file 12896_2018_488_MOESM4_ESM.docx]
